# Mechanism of elevated LH/FSH ratio in lean PCOS revisited: a path analysis

**DOI:** 10.1038/s41598-024-58064-0

**Published:** 2024-04-08

**Authors:** Gita Pratama, Budi Wiweko, Asmarinah, Indah S. Widyahening, Trinovita Andraini, Hartanto Bayuaji, Andon Hestiantoro

**Affiliations:** 1 Department of Obstetrics and Gynecology, Faculty of Medicine Universitas Indonesia, Dr. Cipto Mangunkusumo General Hospital, Jakarta, Indonesia; 2 Cluster of Human Reproduction, Infertility and Family Planning, Indonesian Medical Education and Research Institute (IMERI), Faculty of Medicine Universitas Indonesia, Jakarta, Indonesia; 3 Yasmin IVF Clinic, Dr. Cipto Mangunkusumo General Hospital, Jakarta, Indonesia; 4 Department of Medical Biology, Faculty of Medicine Universitas Indonesia, Jakarta, Indonesia; 5 Department of Community Medicine, Faculty of Medicine Universitas Indonesia, Jakarta, Indonesia; 6 Department of Physiology, Faculty of Medicine Universitas Indonesia, Jakarta, Indonesia; 7 Department of Obstetrics and Gynecology, Faculty of Medicine Universitas Padjadjaran, Bandung, Indonesia

**Keywords:** Dynorphin, Kisspeptin, Neurokinin B, AMH, FAI, HOMA-IR, LH/FSH ratio, Lean, PCOS, Endocrinology, Reproductive biology, Reproductive disorders, Chemical biology, Metabolic pathways

## Abstract

Polycystic ovary syndrome (PCOS) is the most common endocrine disorder affecting 5–20% of reproductive-age women. However, the treatment of PCOS is mainly based on symptoms and not on its pathophysiology. Neuroendocrine disturbance, as shown by an elevated LH/FSH ratio in PCOS patients, was thought to be the central mechanism of the syndrome, especially in lean PCOS. LH and FSH secretion are influenced by GnRH pulsatility of GnRH neurons in the hypothalamus. Kisspeptin is the main regulator of GnRH secretion, whereas neurokinin B (NKB) and dynorphin regulate kisspeptin secretion in KNDy neurons. This study aims to deepen the understanding of the neuroendocrine disorder in lean PCOS patients and its potential pathophysiology-based therapy. A cross-sectional study was performed at Dr. Cipto Mangunkusumo Kencana Hospital and the IMERI UI HRIFP cluster with 110 lean PCOS patients as subjects. LH, FSH, LH/FSH ratio, kisspeptin, NKB, dynorphin, leptin, adiponectin, AMH, fasting blood glucose, fasting insulin, HOMA-IR, testosterone, and SHBG were measured. Bivariate and path analyses were performed to determine the relationship between variables. There was a negative association between dynorphin and kisspeptin, while NKB levels were not associated with kisspeptin. There was no direct association between kisspeptin and the LH/FSH ratio; interestingly, dynorphin was positively associated with the LH/FSH ratio in both bivariate and pathway analyses. AMH was positively correlated with the LH/FSH ratio in both analyses. Path analysis showed an association between dynorphin and kisspeptin levels in lean PCOS, while NKB was not correlated with kisspeptin. Furthermore, there was a correlation between AMH and the LH/FSH ratio, but kisspeptin levels did not show a direct significant relationship with the LH/FSH ratio. HOMA-IR was negatively associated with adiponectin levels and positively associated with leptin and FAI levels. In conclusion, AMH positively correlates with FAI levels and is directly associated with the LH/FSH ratio, showing its important role in neuroendocrinology in lean PCOS. From the path analysis, AMH was also an intermediary variable between HOMA-IR and FAI with the LH/FSH ratio. Interestingly, this study found a direct positive correlation between dynorphin and the LH/FSH ratio, while no association between kisspeptin and the LH/FSH ratio was found. Further research is needed to investigate AMH and dynorphin as potential therapeutic targets in the management of lean PCOS patients.

## Introduction

Polycystic ovary syndrome (PCOS) is the most common and complex endocrine disturbance in women, affecting 5–20% of women of reproductive age^1^. Its diagnosis according to the Rotterdam consensus criteria fulfilled 2 out of these 3 symptoms: oligoovulation or anovulation, clinical or biochemical hyperandrogenism, and polycystic ovarian morphology on ultrasound, excluding other endocrine and gynecology disturbances^2^. Obesity is one of the factors related to PCOS; however, significant numbers of PCOS patients have a normal body mass index (lean PCOS)^3,4^. It is assumed that the pathophysiology of both phenotypes (obese and lean PCOS) are not the same^5^. Insulin resistance and metabolic syndromes such as hypertension, dyslipidemia, and central obesity occur more often in obese patients with PCOS^3,6,7^. On the other hand, luteinizing hormone (LH) levels and the LH to follicle stimulating hormone (FSH) ratio are significantly higher in lean patients with PCOS^8,9^. This highlights that neuroendocrine disturbances may be the most important mechanism in lean PCOS patients.

PCOS etiology is not well understood, but it is speculated to occur as a result of a complex interaction between genetic and environmental factors^10,11^. Intrauterine hyperandrogen exposure has been proposed as a key factor causing the reprogramming of multiple genes contributing to the development of PCOS, which affects the hypothalamus-pituitary axis and metabolic disturbance, increasing LH secretion and abdominal fat accumulation and elevating insulin resistance risks at puberty and adolescence^12^. LH secretion by the anterior pituitary is affected by gonadotropin releasing hormone (GnRH) pulses produced by GnRH neurons in the hypothalamus. PCOS patients also have decreased FSH, thus increasing the LH/FSH ratio, elevating androgen synthesis from theca cells in the ovarium, and finally causing excess androgen production. Furthermore, this condition will prevent follicular development and chronic anovulation. Small antral follicles accumulate and lead to the formation of polycystic ovarian morphology^13–15^.

Kisspeptin is a potent GnRH neuron regulator, principally involved in establishing the onset of puberty and fertility. The kisspeptin neuron in the infundibular nucleus is called the kisspeptin/neurokinin B/dynorphin (KNDy) neuron, which is essential in the negative feedback of sex hormones to GnRH neurons^16,17^. Therefore, abnormalities in kisspeptin neurons are primarily assumed to contribute to LH hypersecretion in PCOS pathogenesis. However, not all studies found higher kisspeptin levels in PCOS women than in control women^18–23^. Heterogeneity in PCOS phenotypes is suspected to be the underlying cause of this inconsistency.

Dapas et al.^24^ studied data from the Genome Wide Association Studies (GWAS) and found two subtypes of PCOS, the ‘reproductive’ type and the ‘metabolic’ type. ‘Reproductive’ types have high LH and SHBG levels with low insulin levels and BMI, whereas ‘metabolic’ types have the opposite. Interestingly, both subtypes have genetic variations that differ from each other. This finding raises further questions about the mechanism of the increased LH/FSH ratio in PCOS patients, especially in lean PCOS^24^. The management aspect of lean PCOS patients remains unclear. Currently, the first-line management of all phenotypes of PCOS patients is to adopt a healthy lifestyle through diet and exercise. However, it does not seem to significantly improve patients with lean PCOS, especially in relation to ovulation and infertility^25^.

Recent studies have shown that KNDy neurons can be a potential clinical target, essential in regulating GnRH/LH hyperactivity in PCOS as an etiology-based therapy, at least in certain phenotypes of PCOS patients^26,27^. Thus, this study aims to further investigate the association between kisspeptin, neurokinin B (NKB) and dynorphin and other endocrine and metabolic variables with the LH/FSH ratio in lean PCOS patients.

**Figure 1:**
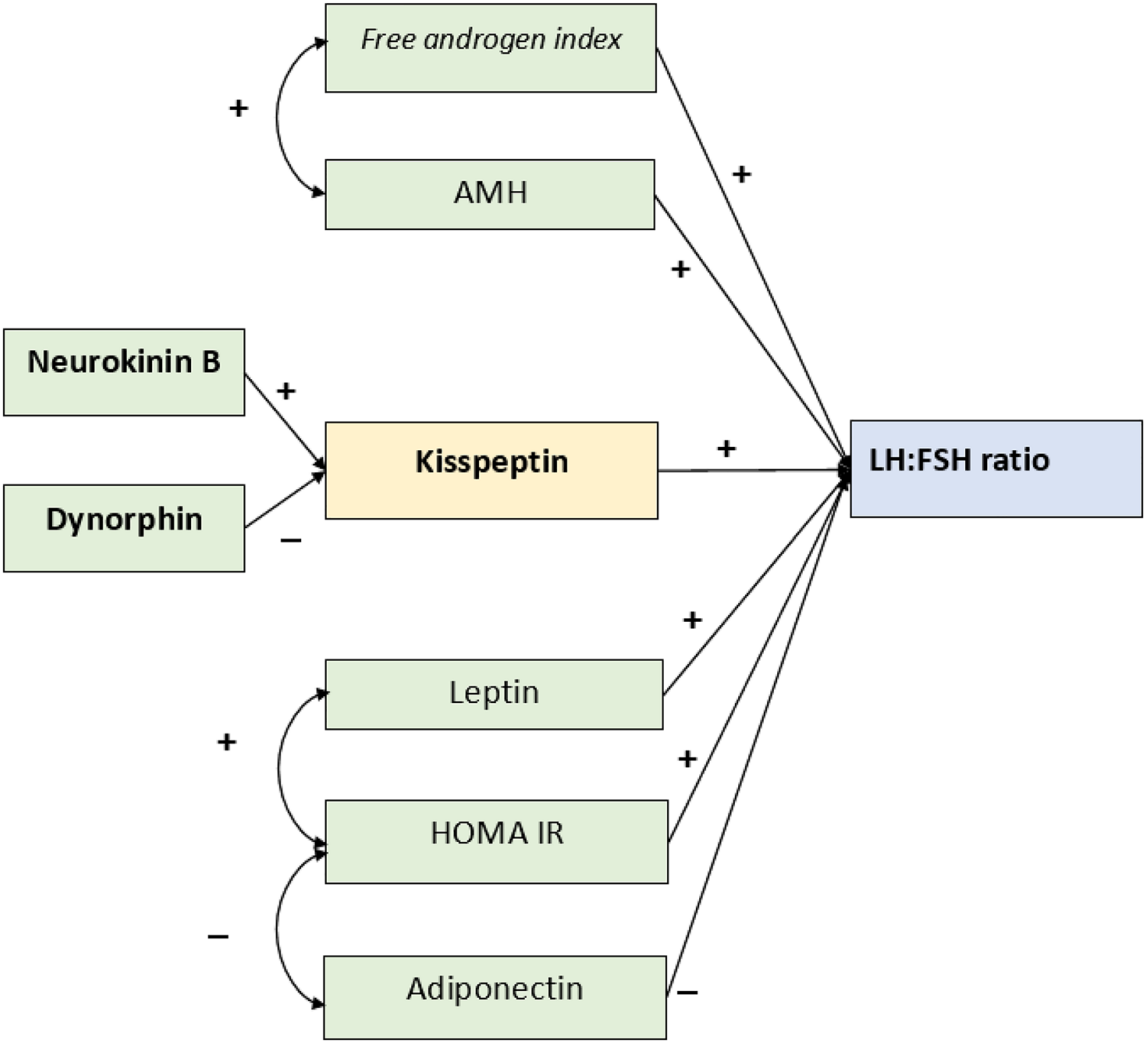
Input diagram for the path analysis of relationships between NKB, dynorphin, AMH, leptin, adiponectin, FAI, and HOMA-IR with kisspeptin and LH to FSH ratio of lean PCOS patients.

The input path diagram figure was created to elucidate the relationship between the variables included in this study. The diagram was constructed based on multiple literature studies. It is hypothesized that NKB and dynorphin regulate kisspeptin secretion from KNDy neurons. Dynorphin has an inhibitory effect and NKB has a stimulatory effect on kisspeptin production^27^. Kisspeptin will bind to GPR54 receptor on GnRH neurons and stimulate GnRH pulsatility and LH secretion from the pituitary consecutively^16,17,19,20,22^. Furthermore, other factors such as androgen, anti-Mullerian hormone (AMH), leptin, and insulin resistance are hypothesized to have a positive impact on the LH:FSH ratio, while adiponectin will decrease the LH:FSH ratio^9,19,20,22,25^ (Fig. 1).

## Material and methods

### Sample collection

PCOS was diagnosed following the Revised 2003 consensus on diagnostic criteria (Rotterdam ESHRE/ASRM-Sponsored PCOS Consensus Workshop Group 2004), which needed meeting any two of the following three criteria: signs of oligo- or anovulation, hyperandrogenism and polycystic ovary morphology identified through ultrasonography. The study was conducted as a cross-sectional examination involving 110 women of reproductive age with PCOS. These participants were recruited from the Reproductive Cluster Yasmin, Dr. Cipto Mangunkusumo Kencana Hospital, Jakarta, Indonesia, between September 2021 and August 2022. Patients with hyperandrogenism or oligomenorrhea due to other causes, such as Cushing’s syndrome, congenital adrenal hyperplasia, hypothyroidism, or elevated serum prolactin levels, were excluded from the study. Additionally, subjects who had taken medications known to affect the HPG axis, such as corticosteroids, hormonal therapy, antiepileptic drugs, or antipsychotic drugs, within the last 6 months were also excluded. Consecutive sampling was used to select the participants until the needed sample size was reached. The enrolled patients underwent thorough physical examinations, ultrasonography, and laboratory tests. The PCOS samples were equally divided based on their body mass index (BMI) following the Asia Pacific criteria, which defines normal weight as a BMI of 18.5–22.9 kg/m^2^ and obesity as a BMI higher than 25 kg/m^2^. Blood samples (5 ml) were collected from all subjects during the follicular phase (until day 5 of the menstrual cycle) after an overnight fast of 10–12 h to ensure accurate results for fasting glucose and fasting insulin levels for HOMA-IR evaluation. The presence of hyperandrogenism was assessed using the Ferriman–Gallwey (FG) score, which measured terminal hair growth on eleven different body areas based on a scale from 0 to 4 according to the FG scoring system and the established cut-off for PCOS in Asia^25^. The free androgen index (FAI), a measure of biologically active testosterone levels in the blood, was calculated by multiplying 100 by the total testosterone level divided by the SHBG level. Insulin resistance was evaluated using the Homeostatic Model Assessment for Insulin Resistance (HOMA-IR).

### Ethical approval

Ethical approval was granted by the Ethics Committee of the Faculty of Medicine, Universitas Indonesia—Dr. Cipto Mangunkusumo Hospital (KET-/197/UN2.F1/ETIK/PPM.00.02/2021). Written informed consent was obtained from each participant. The study followed the ethical standards of the Declaration of Helsinki, revised in 2008, and fulfills the principles of Good Clinical Practice (GCP).

### Measurement of hormone levels

Levels of luteinizing hormone (LH), follicle-stimulating hormone (FSH), total testosterone (T), anti-Müllerian hormone (AMH), fasting insulin, and sex-hormone binding globulin (SHBG) were assessed using TOSOH (Tosoh India Pvt. Ltd., Mumbai, India) following the manufacturer’s protocols. Prior to examination, all samples were thawed, diluted, and placed into 500 µl sample caps. These sample caps, along with the corresponding reagents, were then inserted into the instrument. The process involved filling the diluent, washing the buffer tank, and emptying the waste tank. Subsequently, the software was used to complete the sample identity, and upon pressing the start button on the instrument, the results of hormone levels became available. Adiponectin, leptin, and fasting glucose levels were determined using ELISA kits based on the sandwich ELISA principle for each component.

### Statistical analysis

Demographic and endocrine characteristics are presented as the mean ± SE (standard error). The collected data were normalized using the Kolmogorov‒Smirnov test to assess data distribution. Correlation tests between dependent and independent variables were performed using the Pearson test if both numeric variables showed a normal distribution or the Spearman test if they did not exhibit a normal distribution. A significance level of *P* < 0.05 was considered to indicate statistical significance. The statistical or bivariate analysis was conducted using SPSS version 26.0.

### Path analysis

Path analysis is a valuable statistical technique used to explore potential causal relationships, whether direct or indirect, among a set of variables. Therefore, it is useful to test the theoretical model^28^. Fit indices and acceptable values for these indices were considered as ratio of x^2^ to degrees of freedom (df) ≤ 2, *goodness-of-fit index* (GFI) ≥ 0.95, *comparative fit index* (CFI) ≥ 0.95, *root mean square error of approximation* (RMSEA) < 0.06, *normed fit index* (NFI) ≥ 0.95, *incremental fit index* (IFI) ≥ 0.95, and *Tucker Lewis index* (TLI) ≥ 0.95. Path analysis was performed using JASP software, version 0.17.2.1.

## Results

The demographic and endocrine characteristics of a total of 110 lean women with PCOS are presented in Table 1. The mean age of the women in this study was 25.74 years (SD = 3.08). The average body weight of the subjects reached an average of 52.96 kg (SD = 5.81), with a median BMI of 21.8 kg/m^2^ (16–24). The subject’s average fasting glucose level was 93.68 mg/dL (SD = 11.57), the median fasting insulin level was 6.05 µIU/mL (1.1–23.6), and the median HOMA-IR was 1.41 (0.24–5.56), which indicated that the majority of subjects did not have insulin resistance. Testosterone levels of 62.74 (10.94–212.59), SHBG 60.57 (22.31–492.52), FAI 3.67 (0.25–22.52) and FG score of 6 (1–13) correspond to hyperandrogenism for Indonesian women^29^. The average LH level was 11.07 ± 5.44, and the FSH level was 6.54 ± 2.68, with an LH/FSH ratio of 1.74 (SD = 0.60). The median levels of kisspeptin, dynorphin, and NKB were 23.77 (12.79–439.74), 158.78 (14.03–1059.81), and 420.14 (33.17–2668.78), respectively, while the median levels of leptin and adiponectin were 3.79 (0.53–10.90) and 5.14 (1.44–17.50), respectively.

**Table 1:** Characteristics of lean PCOS patients.

Characteristics	Results
Age (years)	25.74 ± 3.08
BMI (kg/m^2^)	21.8 (16–24)
Weight (kg)	52.96 ± 5.81
Waist circumference (cm)	73.52 ± 5.38
Testosterone (ng/dL)	62.74 (10.94–212.59)
SHBG (nmol/L)	60.57 (22.31–492.52)
FAI	3.67 (0.25–22.52)
Ferriman–Gallwey score	6 (1–13)
Fasting glucose (mg/dL)	93.68 ± 11.57
Fasting insulin (uIU/mL)	6.05 (1.1–23.6)
HOMA-IR (%)	1.41 (0.24–5.56)
AMH (ng/mL)	11.80 (2.06–39.94)
FSH (mIU/mL)	6.54 ± 2.68
LH (mIU/mL)	11.07 ± 5.44
LH/FSH ratio	1.74 ± 0.60
Leptin (ng/mL)	3.79 (0.53–10.90)
Adiponectin (ng/mL)	5.14 (1.44–17.50)
Kisspeptin (ng/mL)	23.77 (12.79–439.74)
Dynorphin (pg/mL)	158.78 (14.03–1059.81)
NKB (pg/mL)	420.14 (33.17–2668.78)

Relationships between NKB, dynorphin, and other hormonal and metabolic parameters and the kisspeptin levels of lean PCOS patients are shown in Table 2. A significant relationship was found between dynorphin and kisspeptin levels (*P* = 0.001); however, there was no relationship between NKB and kisspeptin levels (*P* = 0.268). Testosterone (*P* = 0.049) and SHBG (*P* = 0.048) levels were also shown to have a significant relation with the kisspeptin levels of lean PCOS patients.

**Table 2:** Relationship between chosen parameters and kisspeptin in lean PCOS patients.

Parameters	Estimation	95% Confidence interval		*P* value*
		Lower bound	Upper bound	
NKB	− 0.101	− 0.279	0.078	0.268
Dynorphin	− 0.294	− 0.472	− 0.116	**0.001**
Testosterone	1.045	0.008	3.753	**0.049**
SHBG	− 1.072	− 3.879	− 0.018	**0.048**
FAI	− 1.497	− 3.638	0.033	0.054
FG score	0.127	− 0.155	0.713	0.205
Fasting glucose	0.091	0.803	2.234	0.352
Fasting insulin	4.913	− 6.287	22.244	0.270
HOMA-IR	− 5.061	− 22.121	6.334	0.273
AMH	− 0.099	− 0.520	0.194	0.368
FSH	0.019	− 0.350	0.423	0.852
LH	0.466	− 0.414	2.011	0.194
LH/FSH ratio	0.036	− 0.469	0.653	0.745
Leptin	− 0.188	− 0.649	0.050	0.092
Adiponectin	0.052	− 0.301	0.490	0.636
BMI (kg/m^2^)	0.036	− 0.626	0.848	0.767
Weight (kg)	− 0.104	− 1.022	0.422	0.411
Waist circumference (cm)	0.027	− 0.539	0.705	0.791

*Linear regression test.

Significant values are in bold.

Relationships between NKB, kisspeptin, dynorphin, and other metabolic and hormonal parameters and the LH to FSH ratio of lean PCOS patients are shown in Table 3. Similar to previous results, a significant relationship was found between dynorphin levels and the LH to FSH ratio (*P* = 0.020); however, there was no relationship between kisspeptin (*P* = 0.275) and NKB (*P* = 0.214) levels and the LH to FSH ratio. AMH levels were also found to have a significant relation with the LH to FSH ratio (*P* = 0.001) of lean PCOS patients.

**Table 3:** Relationship between chosen parameters and LH to FSH ratio in lean PCOS patients.

Parameter	Estimation	95% Confidence interval		*P* value*
		Lower bound	Upper bound	
NKB	− 0.053	− 0.169	0.038	0.214
Dynorphin	0.208	− 0.033	0.384	**0.020**
Kisspeptin	− 0.049	− 0.196	0.056	0.275
Testosterone	− 0.053	− 1.321	1.050	0.821
SHBG	0.044	− 1.109	1.336	0.854
FAI	0.028	− 1.114	1.209	0.935
FG score	0.024	− 0.197	0.346	0.588
Fasting glucose	− 0.217	− 0.053	0.030	0.590
Fasting insulin	− 1.174	− 11.597	6.185	0.547
HOMA-IR	1.191	− 6.230	11.504	0.556
AMH	0.321	0.147	0.496	**0.001**
Leptin	− 0.013	− 0.250	0.190	0.788
Adiponectin	0.162	− 0.017	0.340	0.076
BMI (kg/m^2^)	0.087	− 0.180	0.387	0.470
Weight (kg)	− 0.038	− 0.321	0.236	0.764
Waist circumference (cm)	− 0.028	− 0.275	0.208	0.784

*Linear regression test.

Significant values are in bold.

**Figure 2:**
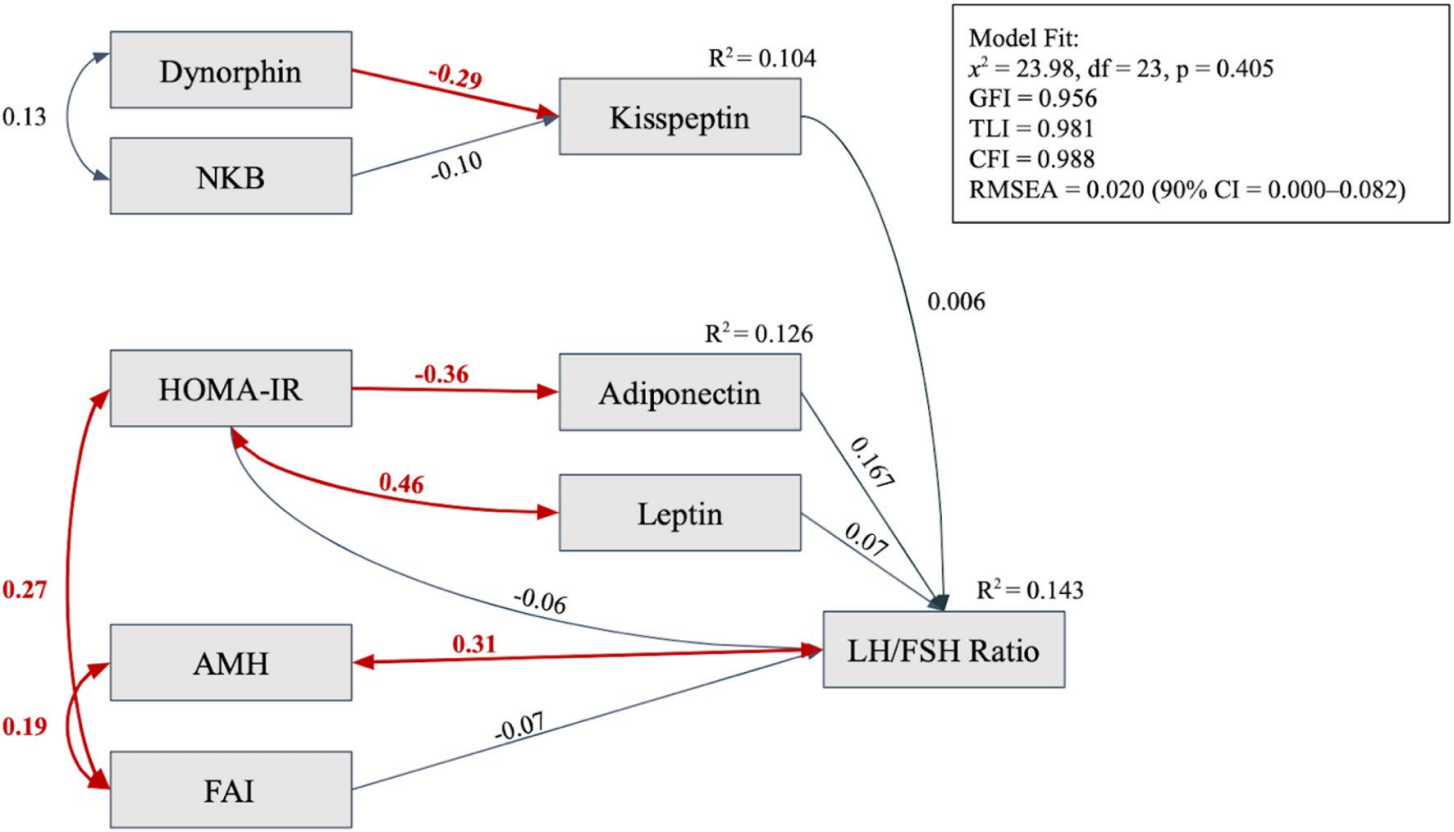
Path analysis of relationships between NKB, dynorphin, AMH, leptin, adiponectin, FAI, and HOMA-IR with kisspeptin and LH to FSH ratio of lean PCOS Patients. One-way arrows indicate a regression relationship; Two-way arrows indicate a correlation relationship; The red arrows indicate a significant relationship between the two variables with the numbers above the arrows as estimations of the relationship.

A path analysis based on the modified model is drawn in a diagram in Fig. 2, showing relationships between dynorphin and NKB with kisspeptin levels and between kisspeptin, leptin, adiponectin, AMH, FAI, and HOMA-IR with the LH to FSH ratio. Single-headed arrows in the diagram represent direct influences, while wires or slings denote covariance or correlations. Tables 4 and 5 express each variable’s regression and correlation coefficients. Before the path analysis was performed, data transformation with Log10 was concluded to turn the data distribution normal as a requirement for path analysis.

**Table 4:** Regression analysis of the modified model.

Predictors	Response variables	Estimation	95% Confidence interval		*P* value*
			Lower bound	Upper bound	
NKB	Kisspeptin	− 0.101	− 0.279	0.078	0.268
Dynorphin	Kisspeptin	− 0.294	− 0.472	− 0.116	**0.001**
FAI	LH/FSH ratio	0.012	− 0.170	0.194	0.899
Kisspeptin	LH/FSH ratio	0.052	− 0.122	0.225	0.558
Leptin	LH/FSH ratio	0.097	− 0.092	0.285	0.315
Adiponectin	LH/FSH ratio	0.171	− 0.005	0.347	0.057
HOMA-IR	LH/FSH ratio	− 0.082	− 0.290	0.127	0.443
Dynorphin	LH/FSH ratio	0.223	0.050	0.396	**0.012**
HOMA-IR	Adiponectin	− 0.358	− 0.534	− 0.182	** < 0.001**

Significant values are in bold.

**Table 5:** Correlations in the path analysis of the modified model.

Variable 1	Variable 2	Estimation	95% Confidence interval		*P* value*
			Lower bound	Upper bound	
AMH	LH/FSH ratio	0.313	0.133	0.493	** < 0.001**
NKB	Dynorphin	0.129	− 0.057	0.316	0.174
Leptin	HOMA-IR	0.479	− 0.274	− 0.685	** < 0.001**
FAI	AMH	0.146	− 0.042	0.333	**0.041**
FAI	HOMA-IR	0.266	0.098	0.434	**0.002**

Significant values are in bold.

The path analysis showed a significant relationship between dynorphin and kisspeptin levels in lean PCOS patients (*P* = 0.001) but not between NKB and kisspeptin levels (*P* = 0.268). A correlation was found between AMH and the LH to FSH ratio (*P* < 0.001); however, kisspeptin did not show any relationship with the LH to FSH ratio (*P* = 0.558). HOMA-IR was significantly related to adiponectin levels (*P* < 0.001), leptin levels (*P* < 0.001) and FAI (*P* = 0.002). AMH also correlated significantly with FAI (*P* = 0.041), other than the LH to FSH ratio.

This path analysis fulfilled the Fit Index criteria with a x^2^ value of 23.98 (df = 23, *P* = 0.405), which is divided by the degree of freedom (df), resulting in a value below two (23.98/23 = 1.043), Goodness-of-Fit Index (GFI) 0.971, Comparative Fit Index (CFI) 1.000, Root Mean Square Error of Approximation (RMSEA) 0.000, Normed Fit Index (NFI) 0.868, Incremental Fit Index (IFI) 1.062, and Tucker Lewis Index (TLI) 1.126. The values above indicate that the overall fit index is acceptable and is considered the best fit for the final model. All conclusions in this study were drawn using a modified model. The coefficient *R*^2^, which describes the efficiency and fitness of the final model, is 0.143 for the LH/FSH ratio and 0.104 for kisspeptin. This means that the model can explain the 14.3% difference in the value of the LH/FSH ratio and the 10.4% difference in the kisspeptin value.

## Discussion

Approximately 20–50% of women with PCOS have a normal body weight (lean), and it is presumed that the pathophysiology of the phenotype differs from obese PCOS patients^5^. Insulin resistance and metabolic syndrome, such as hypertension, central obesity, and dyslipidemia, are more prevalent in obese PCOS patients^3,6,7^. Studies have shown that LH levels and the LH/FSH ratio are significantly higher in lean PCOS women than in obese women ^8,9^. These findings suggest that neuroendocrine disturbances may be the most crucial mechanism in lean PCOS patients. Some studies have found a negative correlation between LH levels, LH/FSH ratio, and BMI^8,30^. Moreover, other research has demonstrated correlations between increased LH, androgen levels, and insulin levels^30,31^. Anti-Mullerian hormone (AMH) levels positively correlate with increased LH in PCOS patients^32,33^, but there are currently no data on the association of these parameters with an increased LH/FSH ratio, particularly in the lean PCOS population.

### Bivariate analysis

In this study, a significant negative relationship was observed between dynorphin levels and kisspeptin levels (Table 2). This finding aligns with previous research, which suggested that dynorphin acts as an inhibitor of kisspeptin secretion in KNDy neurons. Animal studies have indicated that the impact of dynorphin on kisspeptin secretion occurs through two mechanisms: direct inhibition of KNDy neurons and direct influence on GnRH neurons via KOR receptors. A crucial role of dynorphin is its responsibility in terminating GnRH pulsatility^34–36^. On the other hand, in this study, NKB levels were not significantly associated with kisspeptin (Table 4). NKB acts on NK3R receptors in KNDy neurons and is considered a primary stimulator of kisspeptin secretion. Administration of senktide (a selective NK3R agonist) can stimulate LH, although not as potently as kisspeptin in animal experiments^37^. However, similar to our findings, some studies have also not demonstrated a significant relationship between NKB and kisspeptin. Intravenous administration of NKB in healthy men and women did not increase LH and FSH levels^38^.

A significant relationship was observed between testosterone and kisspeptin levels (Table 2). It is hypothesized that hyperandrogenic conditions lead to disturbances in the ovarian steroid hormone feedback mechanism, particularly estradiol and progesterone, in the hypothalamus. Experimental studies on various animal models have shown that prenatal androgen exposure can induce PCOS phenotypes characterized by insulin resistance and increased LH levels^39^. Additionally, Osuka et al. demonstrated that prenatal (rather than postnatal) dihydrotestosterone (DHT) administration increased kisspeptin expression in the arcuate nucleus of rats^40^.

Interestingly, this study did not find a significant association between kisspeptin levels and the LH/FSH ratio in lean women with PCOS (Table 3). This finding contrasts with previous studies demonstrating that exogenous kisspeptin administration can increase LH secretion and pulsatility^41,42^. However, Daghestani et al.^43^ also did not find an increase in kisspeptin levels in lean women with PCOS compared to controls, despite an elevated LH/FSH ratio. Katulski et al.^44^ discovered a correlation between kisspeptin pulsatility and episodic LH secretion in women with PCOS and regular menstrual cycles (eumenorrhea). However, in women with PCOS and oligomenorrhea (menstrual interval > 45 days), no correlation was found between kisspeptin levels and LH pulsatility. This phenomenon suggests that PCOS involves neuroendocrine disturbances, specifically alterations in kisspeptin pulsatility patterns, leading to disrupted LH pulsatility and anovulation. This may explain the lack of association between kisspeptin levels and the LH/FSH ratio in this study, as the absence of increased kisspeptin levels in lean women with PCOS experiencing oligomenorrhea may be due to changes in its pulsatility pattern. Romero-Ruiz et al.^45^ also demonstrated that exogenous kisspeptin administration can improve folliculogenesis and trigger ovulation in women with PCOS. Out of 12 women with PCOS administered GMP-grade kisspeptin 54 twice daily for 21 days, three subjects experienced follicular development, and two of them underwent ovulation. Exogenous kisspeptin administration may stimulate folliculogenesis by improving the synchronization of kisspeptin and LH pulsatility patterns.

This study revealed a significant positive correlation between AMH levels and the LH/FSH ratio in lean women with PCOS (Table 3). Several previous studies have reported similar findings, demonstrating a positive correlation between AMH and LH levels in women with PCOS^46–48^. In women with PCOS, there is an increased production of AMH from ovarian granulosa cells compared to controls. Excessive AMH activity in granulosa cells leads to decreased aromatase enzyme expression, inhibiting folliculogenesis and ovulation and resulting in follicular arrest. As the number of arrested follicles increases, AMH levels further increase. AMH is believed to act on receptors in GnRH neurons, increasing the LH/FSH ratio^49^. Apart from its ovarian influence, AMH is also thought to directly impact GnRH neuron activity by modulating GnRH secretion and enhancing the sensitivity of gonadotropin-secreting cells in the pituitary for LH secretion. The increased frequency of GnRH and LH can trigger the upregulation of AMH receptor (AMHR2) expression in the pituitary, subsequently enhancing the activity of the LH-ß gene promoter and increasing LH levels. AMH receptor type 2 (AMHR2) has been found in both GnRH neurons and the anterior pituitary^50^. Another study on a PCOS animal model induced with AMH showed increased kisspeptin expression in the hypothalamus, similar to animals administered DHT^40^. This study is the first to discover a positive correlation between AMH levels and the LH/FSH ratio in lean women with PCOS. These results confirm the role of AMH as a neuroendocrine regulator influencing the LH/FSH ratio in PCOS, especially in lean PCOS patients^51^.

This study found no significant associations between insulin levels or HOMA-IR and the LH/FSH ratio (Table 3). This contrasts with a study by Malini and Roy, which showed a positive correlation between insulin resistance and LH levels or the LH/FSH ratio^52^. However, Patel et al.^53^ did not find a correlation between insulin levels and insulin resistance with increased LH. Negative correlations between insulin and LH were found in studies by Lawson et al.^54^ and Banaszewska et al.^55^, with exogenous insulin administration also lowering LH levels. It is known that insulin can affect LH function in theca cells, promoting androgen production. However, the mechanism through which this occurs, whether through increased androgen levels first or not, remains to be seen. The subjects in this study were lean PCOS patients with lower insulin levels and HOMA-IR than obese patients, which may explain the lack of a significant association between HOMA-IR and the LH/FSH ratio. Some studies also found a negative correlation between IMT and the LH/FSH ratio in PCOS, but the mechanisms behind these findings still need to be elucidated^56,57^.

This study found no significant associations between hyperandrogenic parameters, namely, testosterone levels, FG scores, and FAI, and the LH/FSH ratio (Table 3). A study by Abbott et al.^12^ in an animal model demonstrated that administering testosterone to pregnant monkeys increased LH levels in their offspring during puberty compared to those without exposure. However, similar to the results of this study, Khan et al.^57^ did not find a correlation between androgens and LH levels. It is speculated that hyperandrogenic conditions in PCOS may reduce hypothalamic sensitivity to negative feedback from estradiol and progesterone, leading to increased LH secretion and decreased FSH secretion from the pituitary^58,59^. High androgen exposure during intrauterine fetal development can cause structural and functional changes in various neurons’ synapses with GnRH neurons and may persist into adulthood, termed developmental programming^39^. This theory might explain the lack of correlation between androgen levels and the LH/FSH ratio in lean PCOS in this study, where increased androgen levels may not influence structural and physiological changes in the hypothalamus, resulting in GnRH neuron hyperactivity.

### Path analysis

The output path analysis diagram of this study (Fig. 2), to certain extent, differs from the input path diagram. Similar to the hypothesis, dynorphin have a negative relationship to kisspeptin level. AMH has a positive correlation effect to LH/FSH ratio in line to a positive regression relationship as noted in the input diagram. Several additional association between parameters were found and added to the output diagram calculated using modification indices available in the path analysis software (JASP). An example of these additions is the association between insulin resistance (HOMA-IR) and hyperandrogenism (FAI) which was not included in the input diagram. These association were still relevant to the hypothesized pathomechanism in PCOS. Several relationships between variables in the input path diagram are not statistically significant. These results can be caused by a multitude of factors, such as confounding factors in the analyses. The findings in the path analysis were consistent with the bivariate analysis, showing a significant negative relationship between dynorphin and kisspeptin levels, no significant correlations between NKB and kisspeptin or between kisspeptin and the LH/FSH ratio and a significant positive correlation between AMH levels and the LH/FSH ratio. Additionally, the path analysis revealed significant relationships between several other variables, namely, a positive correlation between HOMA-IR and leptin, a negative relationship between HOMA-IR and adiponectin, a positive correlation between HOMA-IR and FAI, and a positive correlation between FAI and AMH. These relationships between variables are correctly predicted in the input path diagram. From these analyses, it can be assumed that the impact of insulin resistance (HOMA-IR) and hyperandrogenism (FAI) on the increase in the LH/FSH ratio in lean PCOS patients occurs through the elevation of AMH levels as an intermediary variable rather than through direct effects. This could explain the absence of a significant association between HOMA-IR or FAI and the LH/FSH ratio.

In PCOS, dysfunction in adipose tissue is believed to lead to insulin resistance and chronic low-grade inflammation. Increased cytokines, such as IL-6 and TNF, can decrease glucose transporter-4 (GLUT-4) expression and impair insulin’s ability to transport glucose. Furthermore, chronic low-grade inflammation is thought to reduce adiponectin levels, an adipokine essential in energy homeostasis regulation, leading to a pro-inflammatory condition and insulin resistance^60,61^. In hyperandrogenism, adipocytes undergo hypertrophy, hypoperfusion, hypoxia, and apoptosis. Subsequently, macrophage recruitment occurs, and adipose tissue releases various inflammatory cytokines believed to cause insulin resistance in a chronic setting^61^. Insulin resistance and hyperinsulinemia also increase androgen levels by promoting progesterone conversion to androgen in ovarian theca cells and decreasing SHBG levels in the liver, increasing free testosterone.

Interestingly, this study found a positive association between dynorphin levels and the LH/FSH ratio, both in the bivariate and path analyses. Apart from being released in KNDy neurons, studies by Weems et al.^35^ suggest that dynorphin is also secreted directly into GnRH neurons. The direct effects of dynorphin on GnRH neurons may accelerate GnRH pulsatile frequency, ultimately leading to excessive LH release, which could cause imbalances in the LH/FSH ratio. Some prior research indicates that naltrexone, an antagonist or weak partial agonist of opioid receptors (considering dynorphin’s action through opioid K/KOR receptors), has been beneficial in managing PCOS. Studies suggest that naltrexone results in weight reduction and is closely associated with LH and LH/FSH ratio reduction in clomiphene-resistant PCOS women, while enhancing ovulation rates when combined with pulsatile GnRH infusion^62–64^. Although the exact relationship between dynorphin and the increased LH/FSH ratio is yet to be elucidated, this study assumes the possibility that dynorphin’s direct positive effect on GnRH neurons significantly influences changes in the LH/FSH ratio.

In sheep and cattle studies, dynorphin was found to colocalize with progesterone receptors in the preoptic area, anterior hypothalamus, and arcuate nucleus, indicating that negative feedback from progesterone is likely mediated through dynorphin secretion from KNDy neurons in these regions^65,66^. In PCOS patients, chronic anovulation leads to decreased progesterone levels, which might result in reduced dynorphin secretion, leading to increased kisspeptin and GnRH secretion and eventually increasing the LH/FSH ratio, as observed in this study. One potential approach to reducing the LH/FSH ratio is to increase dynorphin levels, which can be attempted through progesterone administration or using dynorphin receptor agonists. Moreover, it is hypothesized that there is increased pituitary sensitivity to GnRH in PCOS. PCOS patients administered exogenous GnRH exhibit higher LH secretion than controls. Other studies have shown a correlation between insulin circadian concentration patterns and LH in PCOS patients, indicating a positive correlation^67^. However, in this study, we did not find a correlation between insulin and the LH/FSH ratio, possibly because most lean PCOS patients in this study did not experience insulin resistance.

Based on the path analysis, potential pathophysiology-based therapies for lean PCOS patients include targeting dynorphin, which is expected to reduce kisspeptin and the LH/FSH ratio. Second, drugs with dynorphin agonistic or antagonistic properties could influence the LH/FSH ratio in lean PCOS patients. Third, efforts to reduce AMH levels to physiological conditions might mitigate the suppressive effects of AMH on aromatase enzymes and improve granulosa cell sensitivity to FSH, potentially leading to folliculogenesis and ovulation in lean PCOS women. Since there are currently no drugs acting as AMH antagonists, further research is needed to develop medications that can lower AMH levels in PCOS, particularly in lean PCOS.

### Proposed mechanism

**Figure 3:**
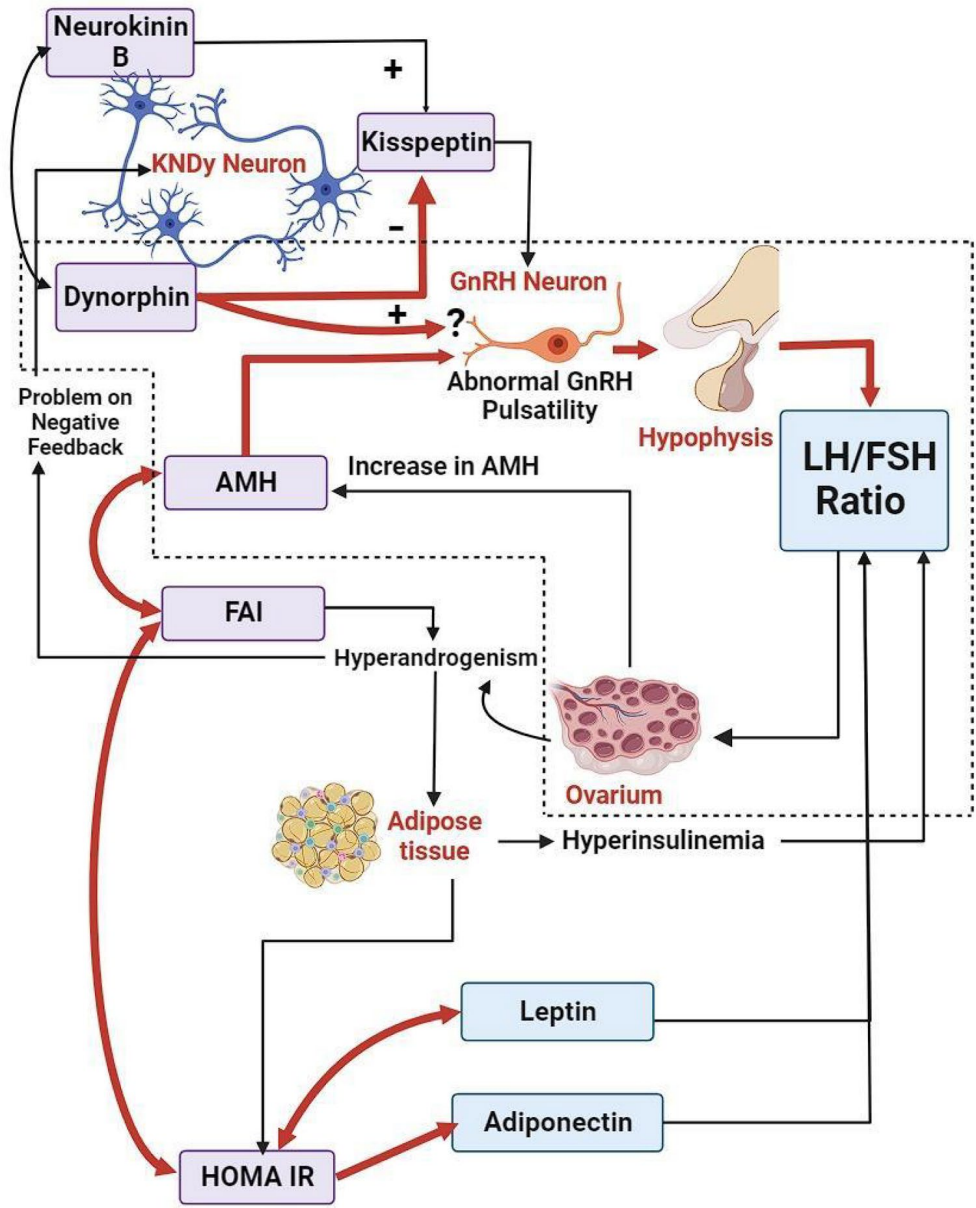
Proposed mechanism or pathophysiology of increased LH/FSH ratio in lean PCOS patients. Dynorphin from KNDy neurons increases the activity of GnRH neurons, increasing GnRH frequency, increasing LH secretion, and increasing the LH/FSH ratio. AMH produced by the ovaries affects GnRH neurons and increases the LH/FSH ratio. HOMA-IR will increase FAI, while FAI will increase AMH. Dot line: pathophysiology of increased LH/FSH ratio in lean PCOS patients. Red arrow: significant relationships found in this study. Black arrow: significant relationships found in other studies.

This study proposed a novel mechanism or pathophysiology of the increased LH/FSH ratio in lean PCOS patients, as shown in Fig. 3.

1. The hyperandrogenic condition in lean PCOS patients leads to disruptions in the negative feedback mechanism of estradiol and progesterone, particularly in KNDy neurons.
2. An increase in NKB levels and a decrease in dynorphin levels result in elevated kisspeptin secretion from KNDy neurons.
3. The increased kisspeptin levels enhance the activity of GnRH neurons, leading to an upsurge in GnRH pulsatile frequency.
4. The direct impact of dynorphin on GnRH neurons is hypothesized to be reduced due to low progesterone levels (further research is warranted).
5. Increased LH and decreased FSH levels from the anterior pituitary cause an elevation in the LH/FSH ratio.
6. Enhanced androgen production from ovarian theca cells occurs due to increased LH levels, and decreased FSH leads to folliculogenesis arrest, accumulating small antral follicles and ultimately increasing AMH levels.
7. AMH binds to the AMHR2 receptor in GnRH neurons, increasing GnRH pulsatile frequency, LH secretion from the anterior pituitary, and the LH/FSH ratio. Additionally, AMH suppresses the expression of aromatase enzymes in granulosa cells, reducing the conversion of androgens to estrogens. Both mechanisms ultimately lead to increased androgen levels.
8. Hyperandrogenic conditions are likely to cause dysfunction in adipose tissue. Adipocytes undergo hypertrophy, hypoperfusion, hypoxia, and apoptosis, releasing various inflammatory cytokines that contribute to insulin resistance. Additionally, the pro-inflammatory adipokine leptin increases production, while the anti-inflammatory adipokine adiponectin decreases production.
9. Insulin resistance leads to hyperinsulinemia, which also increases androgen levels by promoting androstenedione production in ovarian theca cells and reducing SHBG levels in the liver, resulting in elevated free testosterone (FAI) levels.

There are certain limitations due to the absence of parameters that might have significant implications in the pathophysiology of lean PCOS patients, such as GABA neurotransmitters, GPR54 receptors, NK3R, and KOR. Further research endeavours are imperative to explore the therapeutic potential of dynorphin in managing lean PCOS patients. To this end, investigations could employ GnRH neuronal cell lines exposed to dynorphin, with measurements of GnRH levels in the culture medium before and after exposure. Alternatively, experimental animal models administered dynorphin could be utilized, and LH levels could be measured before and after administration. Moreover, the development of drugs with agonistic properties toward dynorphin receptors and antagonistic properties toward AMH receptors is essential to assess their therapeutic potential in lean PCOS patients.

## Conclusion

In conclusion, AMH is directly connected with the LH/FSH ratio, highlighting its significance in the neuroendocrinology of lean PCOS. Path analysis revealed that AMH acted as an intermediary variable between HOMA-IR and FAI, influencing the LH/FSH ratio. Notably, this investigation observed a positive correlation between dynorphin and the LH/FSH ratio, whereas no link was identified between kisspeptin and the LH/FSH ratio. Additional research is needed to explore the potential of AMH and dynorphin as therapeutic targets in the treatment of lean PCOS patients.

## Data Availability

The authors confirm that the data analyses or generations which support the findings of this research are available within the article.
